# Linear Amphiphilic P(BzMA-co-DMAEMA) Statistical Copolymers: Synthesis via RAFT Polymerization and Formation of Nanoassemblies in Aqueous Media

**DOI:** 10.3390/polym18111278

**Published:** 2026-05-22

**Authors:** Stamatios Amarantos, Michaila Akathi Pantelaiou, Aleksander Forys, Barbara Trzebicka, Stergios Pispas

**Affiliations:** 1 Theoretical and Physical Chemistry Institute, National Hellenic Research Foundation, 48 Vassileos Constantinou Ave., 11635 Athens, Greece; stamatis.amarantos@gmail.com (S.A.); apant@eie.gr (M.A.P.); 2 Department of Chemistry, National and Kapodistrian University of Athens, Panepistimiopolis Zografou, 15771 Athens, Greece; 3 Centre of Polymer and Carbon Materials, Polish Academy of Sciences, M. Curie-Sklodowskiej 34, 41-819 Zabrze, Poland; aforys@cmpw-pan.pl (A.F.); btrzebicka@cmpw-pan.pl (B.T.)

**Keywords:** statistical copolymer, amphiphilic copolymer, quaternization, RAFT polymerization, aggregation

## Abstract

Amphiphilic statistical copolymers are valuable synthetic macromolecules for the formation of small, well-defined nanoassemblies able to be utilized as nanocarriers for drug and/or gene delivery applications. In this work, the synthesis of amphiphilic linear statistical copolymers of the poly(benzyl methacrylate-co-dimethylaminoethyl methacrylate) [P(BzMA-co-DMAEMA)] type is described in three different comonomer compositions. Their synthesis was realized through a one-pot reversible addition-fragmentation chain transfer (RAFT) solution polymerization scheme. Further quaternization of the amine groups of DMAEMA with methyl iodide (CH_3_I) resulted in cationic amphiphilic statistical copolymers. Macromolecular characterization was performed using size exclusion chromatography (SEC) and spectroscopic techniques (^1^H-NMR and ATR-FTIR). The aggregation properties of the copolymers in aqueous media were studied via dynamic light scattering (DLS) and electrophoretic light scattering (ELS). Bimodal size distributions were determined in some cases. The BzMA to DMAEMA ratio determined aggregate size, with the copolymer of lower hydrophobic BzMA content producing smaller nanoparticles. Cryogenic transmission electron microscopy (cryo-TEM) showed the presence of spherical assemblies resulting from aggregation of primary micelles in the case of higher BzMA content. The copolymer aggregates experience dissociation at high salt concentration, and the pH-responsiveness of the amine precursors results in the formation of multifunctional potential nanocarriers.

## 1. Introduction

Amphiphilic copolymers are a class of polymeric materials consisting of hydrophobic and hydrophilic monomers. These copolymers can be synthesized in four different comonomer arrangements, which are block, gradient, alternating, or statistical (random) copolymers [1,2]. Lately there has been great interest in synthesizing amphiphilic polymers due to their self-assembly properties through formation of nanoparticles in aqueous solutions. Most of the research that has been done is on block copolymers due to their well-defined microphase separation, in which two large distinct nanodomains are produced by forcing hydrophilic and hydrophobic polymeric blocks into the formed supramolecular nanoassemblies [1,3,4]. On the other hand, the statistical copolymers (in several cases also designated as random copolymers), due to the hydrophilic–hydrophobic monomer topological dispersion within the polymer chain, develop smaller regions, which are affected by monomer ratio and monomer sequence fully coupled to their respective reactivity ratios [2,5,6,7]. This affects their self-assembly by producing smaller aggregates/micelles or even single-chain nanoparticles [8]. In order to produce single-chain nanoparticles, the hydrophilic–lipophilic balance (HLB) plays a vital role [9]. Also, this ratio can determine the morphology of the self-assembled copolymer nanostructures, with larger hydrophobic contents potentially leading to rod-like [10] or even vesicular structures [11]. Amphiphilic statistical copolymers excel over block copolymers due to their time-efficient and convenient one-step synthesis procedure, which marks them as better candidates in industrial applications [6,7,12].

In order to synthesize this class of polymeric materials, controlled radical polymerization (CRP) approaches have been used. Nitroxide-Mediated Radical Polymerization (NMP), Atom Transfer Radical Polymerization (ATRP), and Reversible Addition-Fragmentation Polymerization (RAFT) have been referred to as CRP [6,13]. These techniques combine the simplicity of radical polymerization and their results regarding molecular mass control and chain dispersity corresponding to those of the respective anionic and cationic living polymerizations. Among these three, RAFT and ATRP have gained the most attention due to the plethora of monomers available, good control of macromolecular architecture, molecular mass, low dispersity, and high-end functionality [6,14,15,16]. In this work RAFT polymerization was utilized since the control in macromolecular characteristics and reproducible, reliable, and uniform synthetic outcomes are achieved [17].

Amphiphilic statistical/random copolymers exhibit interesting macromolecular properties enabling their utilization in a variety of applications such as in nanoreactors, membranes, and solid electrolytes for lithium batteries and their biomedical applications such as in tissue engineering, drug delivery, gene therapy, and as antibacterial agents [9,14,18,19,20,21]. In order to achieve the desired application, the specific choice of comonomers is essential. DMAEMA is a monomer carrying a tertiary amine in the side group. From this specific characteristic, a dual-stimuli responsiveness arises, the first being pH-responsiveness, where the protonation and deprotonation of the amine group change chain charge and the polymer chain conformation, and the second being response to temperature changes, as the LCST of PDMAEMA homopolymer is in the range of 35–45 °C [22]. PDMAEMA is of weak cationic nature with a pKa of ca. 7.4 [23], where the developed positive chain charge plays a vital role in gene delivery and bactericidal applications. Furthermore, positive charge presence can be enhanced with a simple quaternization of the tertiary amine group by alkyl halides producing a versatile class of functional polymer materials by the choice of the alkyl group utilized for the quaternization reaction [22,24,25].

On the other hand, BzMA is an alternate candidate for the traditional hydrophobic methyl methacrylate (MMA) used in several applications [12]. BzMA is also a commonly used substitute of the styrene in RAFT polymerization, as it has a better polymerization rate [26]. Because of the aromatic side group, it possesses a relatively high Tg and can form π-π interactions within polymer composites, while its applications can vary based also on its hydrophobicity. PBzMA application can be in orthodontic adhesives, coatings, contact lenses, ionic liquids, and others [14,27].

In a previous study we synthesized and studied amphiphilic hyperbranched P(BzMA-co-DMAEMA) copolymers [28]. In this study, the linear counterparts are successfully synthesized via RAFT polymerization. Three copolymers with different monomer ratios and their chemically modified cationic derivatives are presented. Firstly, the difference in the properties of the hyperbranched and the linear copolymers is annotated. DMAEMA was utilized due to its dual-stimuli responsive character towards pH and temperature changes in aqueous solutions. It is also interesting to investigate how comonomer ratios and comonomer distribution within the polymeric chain affect the solution properties. In the chemically modified counterparts, as the tertiary amine groups of DMAEMA segments are quaternized, a constant cationic charge emerges on the copolymer chains, increasing the water solubility of the copolymers. The P(BzMA-co-DMAEMA) and P(BzMA-co-QDMAEMA) statistical copolymers were studies in terms of their molecular, physicochemical and self-assembly properties and characteristics in aqueous media to understand how these copolymers respond upon varying pH, temperature, ionic strength, and also how they interact with anionic proteins, as is the case with fetal bovine serum proteins in FBS solutions simulating polymer aggregate-protein interactions (or even aggregate-blood serum interactions).

## 2. Materials and Methods

### 2.1. Materials

The monomers benzyl methacrylate (BzMA) and 2-(dimethylamino)ethyl methacrylate (DMAEMA) were obtained from Sigma-Aldrich (Athens, Greece). The monomers were purified by filtering with inhibitor-remover resins (311,332; Sigma-Aldrich, Athens, Greece) packed in glass columns prior to the polymerization. The radical initiator, 2,2-azobis(isobutyronitrile) (AIBN) was recrystallized from methanol before use. The solvent, 1,4-dioxane (≥99.8% pure, Sigma-Aldrich, Athens, Greece), was dried using molecular sieves, while tetrahydrofuran, n-hexane (≥97% pure, Aldrich, Athens, Greece), chain transfer agent, 4-cyano-4-(phenylcarbonothiolylthio) pentanoic acid (CPAD), fetal bovine serum (FBS) phosphate-buffered saline (PBS), and iodomethane (CH_3_I) and all other reagents were used as received from Sigma-Aldrich (Athens, Greece).

### 2.2. Synthesis of P(BzMA-co-DMAEMA) Linear Copolymers

For the synthesis of three linear statistical copolymers with different monomer ratios [30:70], [50:50], and [70:30] of the type P(BzMA-co-DMAEMA), RAFT polymerization was utilized. The synthetic procedure of the representative copolymer LBD1 is detailed below. In a 50 mL single-neck round-bottom flask, the reagents were mixed with a magnetic stirrer. The initiator (AIBN (0.0123 g, 0.75 mmol)), the chain transfer agent CPAD (0.0419 g, 1.5 mmol), the monomers BzMA (0.9 g, 5.1 mmol) and DMAEMA (2.1 g, 13.4 mmol), and lastly the solvent 1,4-dioxane were added to a final volume of 15 mL to achieve 20% *w*/*v* of the produced polymer in the solution. The molar ratio of the CTA:AIBN was adjusted to 2:1 and the monomer weight ratio of BzMA:DMAEMA to 30:70. The flask was sealed with a rubber septum and left to homogenize under stirring while the solution was degassed by nitrogen flow for 20 min. To initiate the polymerization, after degassing, the solution was placed in an oil bath heated to 70 °C and kept under stirring. After 24 h the flask was cooled in the freezer for 20 min and then exposed to atmospheric air to terminate the reaction. The obtained copolymer was precipitated in excess of n-hexane for the removal of the impurities such as unreacted monomers and oligomers. Lastly, the polymer was collected and dried in a vacuum oven for 48 h. All synthesized amine-based copolymers are listed in Table 1 together with their code names and characteristics.

### 2.3. Chemical Modification of P(BzMA-co-DMAEMA) Linear Copolymer

The amphiphilic statistical copolymers were modified in order to quaternize the tertiary amine groups of DMAEMA and produce amphiphilic cationic polyelectrolyte analogues of the copolymers. The LBD1 copolymer (0.5 g, 0.18 mmol) was dissolved in THF to a final volume of 15 mL and kept under stirring. Then, iodomethane (0.208 mL, 0.27 mmol) addition was adjusted to a 1.5:1 molar ratio of iodomethane to polymer amine groups. The flask was wrapped with aluminum foil due to iodomethane’s sensitivity to intense light and kept under moderate stirring for 24 h at room temperature. The final product was collected after evaporation of THF in a rotor evaporator and dried in a vacuum oven for 48 h.

### 2.4. Self-Assembly of Linear Copolymers in Aqueous Solution

Copolymers LBD2 and LBD3 did not dissolve directly in water due to their high hydrophobic content. For these copolymers to self-assemble in a selective solvent, the solvent–nonsolvent protocol was utilized. The polymers (15 mg) were dissolved in THF (1.5 mL), which is an excellent solvent for both copolymer components. Under mild stirring, 15 mL of distilled water was heated at 60 °C, and then the polymer solution in THF was rapidly injected via a syringe. After 2 h the heat was turned off, and the solvent was kept under stirring for 24 h upon completion of the formation of the nanoparticles.

### 2.5. Self-Assembly of Linear Quaternized Copolymers in Aqueous Solution

After quaternization of the amine groups of the polymers, a high positive charge is achieved. This makes the copolymers to be more soluble in aqueous solutions. To prepare solutions for self-assembly studies, the quaternized copolymers (15 mg) were dissolved in 15 mL of water and were heated at 60 °C for 1 h for complete dissolution and equilibration.

### 2.6. Ionic Strength and Fetal Bovine Serum (FBS) Interaction Studies

The ionic strength effect of the amphiphilic polyelectrolyte behavior was investigated with the addition of salt. An exact volume of 1 M NaCl was added to 1 mL of the polymer solution (1 mg/mL) to gradually increase the salt concentration from 0.05 mg/mL to 0.33 mg/mL. Potential interactions of the cationic polymers with serum proteins were investigated by the use of an FBS:PBS solution (1:9 *v*/*v*). In detail, 150 μL of polymer solution in water was added to 1.5 mL of FBS:PBS solution. The solution was allowed to equilibrate for one hour, and then it was analyzed using the DLS technique.

### 2.7. Characterization of the Copolymers

#### 2.7.1. Size Exclusion Chromatography

SEC experiments on the amine copolymers were conducted in the modular instrument consisting of a Waters Model 510 pump (Milford, MA, USA), a Waters Model 600 controller (Milford, MA, USA), a Shimadzu RID-10A refractive index detector (Kyoto, Japan), and a set of four Waters Styragel HR (Milford, MA, USA) series columns with porosity ranging from 500 to 10^6^ Å, at 27 °C. The flow rate of the carrier solvent was 1 mL min^−1^, and chloroform was utilized as the mobile phase. Polystyrene standards with molecular weights between 1000 and 900,000 g mol^−1^ were used for the calibration curve of the instrument.

#### 2.7.2. Proton Nuclear Magnetic Resonance Spectroscopy (^1^H-NMR)

To determine the copolymer composition, ^1^H-NMR spectroscopy measurements were conducted. The solvent used was CDCl_3_, and the copolymer concentration was adjusted to 10 mg/mL. The spectrometer used was the Varian 600 (600 MHz) (Palo Alto, CA, USA) instrument operated by Vjnmr software (openVnmrj rev. 3.2A). Jason software v.6.0.10499 by JEOL JASON (Witney, UK) was used to analyze the spectra.

#### 2.7.3. Attenuated Total Reflectance–Fourier Transform Infrared (ATR-FTIR) Spectroscopy

A single bounce ATR diamond integrated in the Brucker (Billerica, MA, USA) Equinox 55 spectrometer (DuraSamp1IR, SensIR Technologies, Danbury, CT, USA) was used for these measurements. The press was used to record the spectra because the samples were in a solid state. A resolution of 4 cm^−1^ and 64 scans were used for spectra acquisition.

#### 2.7.4. Dynamic Light Scattering (DLS)

DLS studies were carried out using an ALV/CGS-3 compact goniometer system (ALV GmbH, Hessen, Germany) with a JDS Uniphase 22 mW He–Ne laser (ALV GmbH, Hessen, Germany) operating at a 632.8 nm wavelength. The system is equipped with an ALV/LSE-5003 light-scattering electronics unit (ALV GmbH, Hessen, Germany) used for stepper motor drive and limit switch control and an ALV-5000/EPP multi-τ correlator (ALV GmbH, Hessen, Germany) with 288 channels. The obtained autocorrelation functions (and the simultaneously recorded light scattering intensity) were the average of three measurements at a goniometer angle of 90°, analyzed by the cumulants method and the CONTIN algorithm. Size distributions presented in the manuscript are intensity weighted. All aqueous solutions were filtered through 0.45 μm hydrophilic PVDF filters prior to the measurements.

#### 2.7.5. Electrophoretic Light Scattering (ELS)

Zeta potential values, which are directly related to the surface charge of polymer particles in solution, were measured by electrophoretic light scattering experiments conducted on a Nano Zeta Sizer instrument from Malvern (Worcestershire, UK) which is equipped with a 4 mW He–Ne laser operating at 633 nm and at a scattering angle of 173°. Each measurement was an average of 20 repeated scans, and the obtained data were analyzed by the Smoluchowski equation.

#### 2.7.6. Cryogenic Transmission Electron Microscopy (cryo-TEM)

Cryogenic Transmission Electron Microscopy (cryo-TEM) images were obtained using a Tecnai F20 X TWIN microscope (FEI Company, Hillsboro, OR, USA) equipped with a field emission gun and operating at an acceleration voltage of 200 kV. Images were recorded on the Gatan Rio 16 CMOS 4k camera (Gatan Inc., Pleasanton, CA, USA) and processed with Gatan Microscopy Suite (GMS) software version 3.31.2360.0 (Gatan Inc., Pleasanton, CA, USA). Specimen preparation was done by vitrification of the aqueous solutions on grids (grid mesh 200) covered with holey carbon film (Quantifoil R 2/2; Quantifoil Micro Tools GmbH, Großlöbichau, Germany). Prior to use, the grids were activated for 15 s in oxygen plasma using a Femto plasma cleaner (Diener Electronic, Ebhausen, Germany). Cryo-samples were prepared by applying a droplet (3 μL) of the suspension to the grid, blotting with filter paper, and immediately vitrifying in liquid ethane using a fully automated blotting device, Vitrobot Mark IV (Thermo Fisher Scientific, Waltham, MA, USA). After preparation, the vitrified specimens were kept under liquid nitrogen until they were inserted into a cryo-TEM-holder Gatan 626 (Gatan Inc., Pleasanton, CA, USA) and analyzed in the TEM at −178 °C.

## 3. Results and Discussion

### 3.1. Synthesis of Linear P(BzMA-co-DMAEMA)

**Figure 1:**
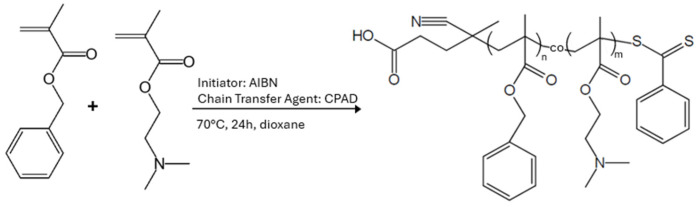
Synthetic scheme for P(BzMA-co-DMAEMA) preparation.

The synthetic procedure of the amphiphilic statistical copolymers was achieved via a one-pot RAFT polymerization synthesis scheme which is displayed in Figure 1. The two monomers were purified with the inhibitor remover resins in columns. The initiator AIBN was utilized in order to effect initiation of the polymerization. CPAD as a chain transfer agent is used to control the propagation of the polymer chains and achieve low polydispersity indices as is considered an effective CTA for methacrylate monomers. As the solvent needs to dissolve both the polymer and the monomers, 1,4-dioxane was utilized. In order to activate AIBN initiator fragmentation and start the polymerization, the solution was heated under stirring in an oil bath at 70 °C. After 24 h, the polymerization was terminated by rapid cooling of the reaction solution and exposure to air. Then, the copolymer was precipitated in excess of n-hexane, which is a poor solvent for the copolymers, to purify it from the byproducts of the synthesis and isolate them in solid form.

### 3.2. Chemical Modification of P(BzMA-co-DMAEMA)

The quaternization of the copolymers happens on the tertiary amine side groups of DMAEMA segments in order to modify the amine polymer and to obtain a cationic amphiphilic polyelectrolyte comonomer with permanent positive charges on the amine groups. For the quaternization of the copolymers, iodomethane (MeI) was utilized as the quaternization agent. The reaction is quantitative and straightforward while the reaction solvent and excess MeI can be easily removed from the product by simple evaporation under medium vacuum since they are both volatile compounds. The quaternized copolymers in solid form were further characterized for their molecular characteristics to be determined.

### 3.3. P(BzMA-co-DMAEMA) and P(BzMA-co-QDMAEMA) Molecular Characterization

**Figure 2:**
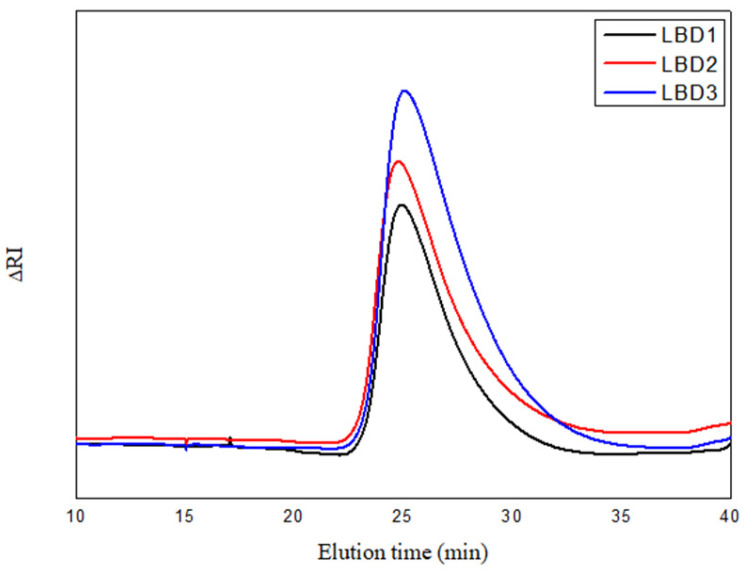
SEC curves of the synthesized amphiphilic copolymers.

Copolymers of the type P(BzMA-co-DMAEMA) with three different comonomer ratios were successfully synthesized and characterized by SEC. The results are presented in Table 1. The copolymers’ molar mass dispersity (Đ = M_w_/M_n_) of 1.53, 1.73, and 1.72 for the samples LBD1, LBD2, and LBD3, respectively, were obtained. From the chromatograms depicted in Figure 2, absorption of the copolymers on the columns may take place, as the curves are not symmetrical and show some tailing at high retention volume. This may be the reason for the polydispersity indices (Đ) determined since they are higher than expected. The average molecular weights illustrated in Table 1 are similar across the samples with values of 3.7, 3.8, and 3.3 × 10^4^ gmol^−1^, respectively. It was designed to have a series of copolymers with similar molar masses but different compositions. Due to the instrument calibration with polystyrene standards, the molecular weights measured are apparent ones.

**Figure 3:**
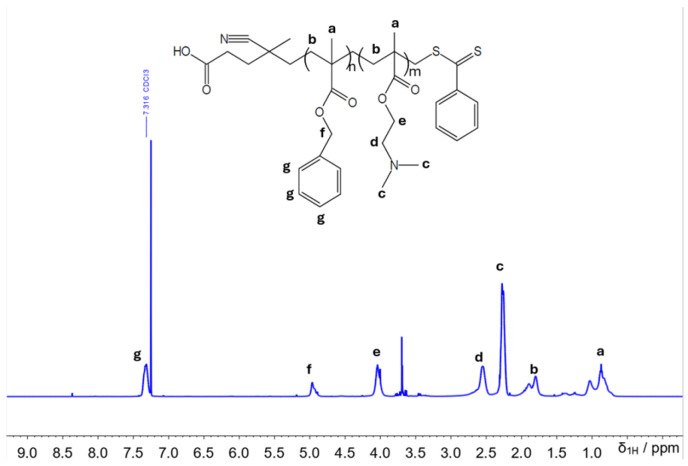
^1^H-NMR spectrum of LBD1 copolymer in CDCl_3_.

The composition in the copolymers was evaluated using ^1^H-NMR spectroscopy. The copolymers were dissolved in CDCl_3_, and compositions were determined by integrating the characteristic f band at 4.9 ppm for BzMA, which corresponds to the methyl group next to the benzene ring, and the d band at 2.6 ppm for DMAEMA, which corresponds to the methyl group next to the amine group. The composition of three polymers can be seen in Table 1. In Figure 3, the spectrum of LBD1 is depicted with the characteristic hydrogen resonance peaks marked. Other spectra can be found in Figures S1 and S2 in the Supporting Information.

**Figure 4:**
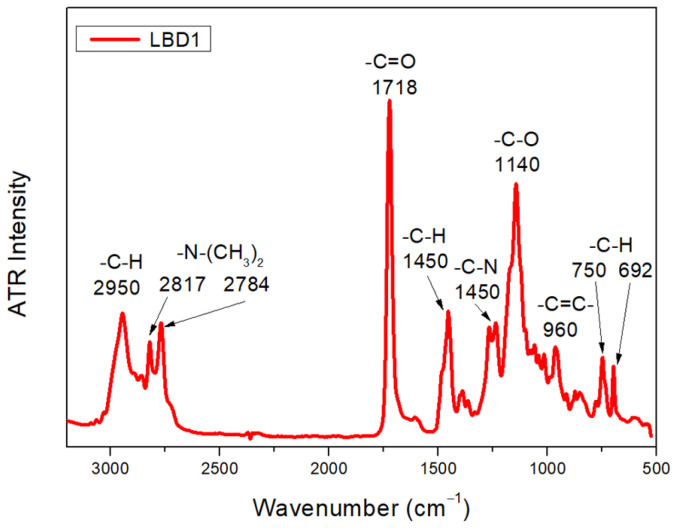
ATR-FTIR spectrum of LBD1 copolymer.

The ATR-FTIR measurements were conducted to confirm the chemical structure of the copolymers. In Figure 4, the ether stretching vibration -C=O at 1718 cm^−1^ is derived from both methacrylic comonomers. The bands at 2817 cm^−1^ and 2784 cm^−1^ indicate the characteristic dimethylamino group stretching of DMAEMA segments [23]. Lastly, the bands at 960 cm^−1^ of -C=C- alkene bending and -C-H out-of-plane bending in 750 cm^−1^ and 692 cm^−1^ refer to the aromatic ring of BzMA segments.

**Figure 5:**
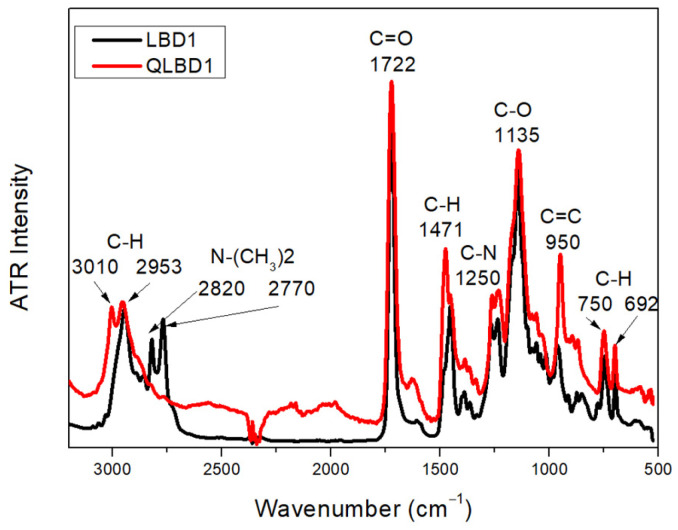
ATR-FTIR spectra of LBD1 and QLBD1 copolymers indicating the successful formation of the quaternized copolymer.

The quaternized amine group cannot be verified directly by ATR-FTIR. Concluding from the absence of the bands at 2817 cm^−1^ and 2784 cm^−1^, corresponding to the -N-(CH_3_)_2_ bonds, the quaternization was successful and nearly quantitative as expected (Figure 5). The results for QLBD2 and QLBD3 can be found in Supplementary Information.

### 3.4. Physicochemical Studies of Linear Copolymers and Their Quaternized Counterparts in Aqueous Media

#### 3.4.1. Self-Assembly of Copolymers in Aqueous Solutions at Neutral pH

**Figure 6:**
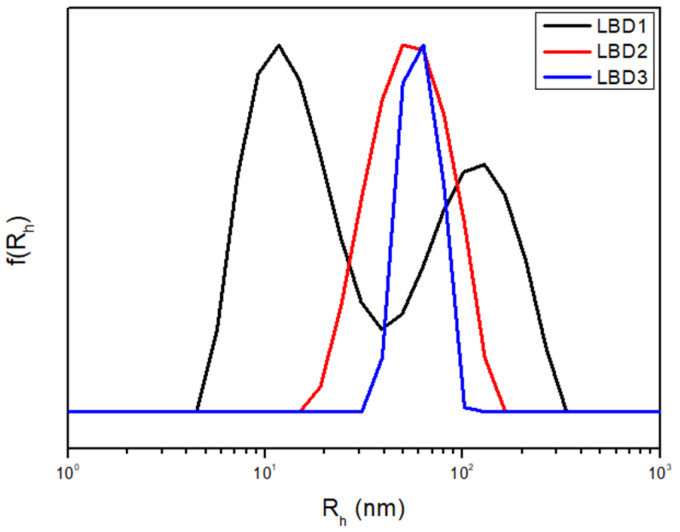
Size distribution from CONTIN analysis of DLS data for LBD copolymers.

Self-assemblies deriving from the copolymers in aqueous solution in neutral pH values (pH ≈ 7) were prepared with the solvent–nonsolvent protocol as detailed earlier. These measurements were conducted by dynamic and electrophoretic light scattering. From the light scattered intensity determined in DLS measurements, it can be concluded that the larger hydrophobic content of the copolymer generates larger aggregates. As the light scattered intensity increases, it indicates the formation of more massive and probably compact structures due to random arrangement of hydrophobic (BzMA) and hydrophilic (DMAEMA) segments within the copolymer chains. For the LBD1 copolymer, two populations appeared in the size distribution profile: one small, corresponding probably to single-chain nanoparticles (SCNs) with R_h_ = 9 nm, and one of large size which may result from the aggregation of the SCNs, showing an average size of R_h_ = 101 nm (Figure 6). This should also be confirmed later by the cryo-TEM measurements. From the DLS spectra (Figure 6) and considering that this technique is sensitive to larger nanoparticles due to its intensity-based nature, the SCNs should be the majority of nanoparticles in the copolymer solution [29]. For copolymers LBD2 and LBD3, the measurements showed a single population with almost identical R_h_ values, thus indicating the formation of primarily multichain nanoparticles in these copolymer solutions. This can be attributed to the high ratio of hydrophobic BzMA monomer segments which as the polymer chains aggregate, form hydrophobic domains within the micelles, due to the polar environment of the aqueous solution. All the nanoparticles were expected to be positively charged, as the DMAEMA segments in the polymer chain are anticipated to be in the outer part of the assemblies (i.e., the particle corona). Unexpectedly, the positive charge of the nanoparticles was not radically affected by the DMAEMA content in the polymer chain (Table 2). This can be attributed to the conformation and arrangement of copolymer chains within the aggregates, with DMAEMA domains being more exposed to the particle surface in the multichain nanoparticles (in the case of LBD2 and LBD3), rather than the large in size aggregates of LBD1, where the BzMA domains may be constrained and shielded better by the polar DMAEMA outer domains (coronas). As for the SCNs, the zeta potential values, which were expected to be more positively charged, cannot be identified because ELS is also an intensity-based technique where the larger nanoparticles are dominating the scattering profile. Lastly, we can compare LBD1 with the previously investigated hyperbranched HB1 copolymer, which is a hyperbranched amphiphilic statistical copolymer with relatively the same composition (17 wt% BzMA and 83 wt% DMAEMA). In the linear form the nanoparticles were smaller, forming the SCNs. In the case of the hyperbranched copolymer, branching introduces conformational constraints that inhibit efficient self-assembly into SCNs, resulting in the formation of larger multichain aggregates with a hydrodynamic radius of 104 nm [28].

**Figure 7:**
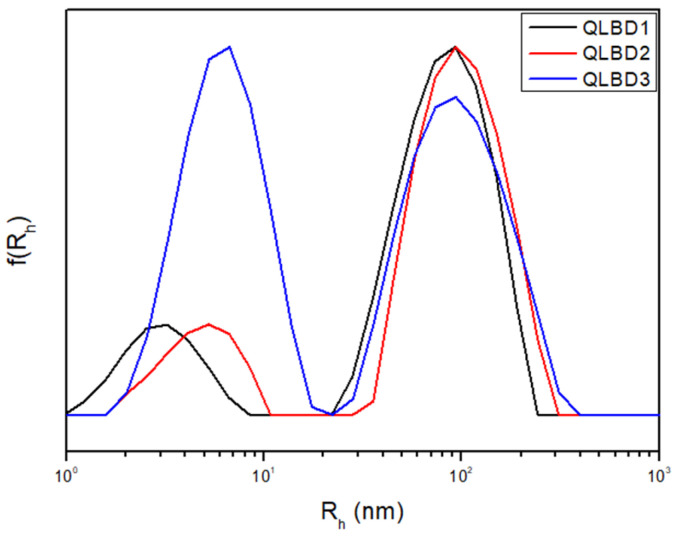
Size distribution from CONTIN analysis of DLS data for quaternized QLBD copolymers.

Particles deriving from the respective quaternized copolymers were prepared with an easy direct dissolution method described earlier. As the QDMAEMA segments in the copolymers have a constant and strongly positive charge, the quaternized copolymers are more hydrophilic, and a more hydrophilic corona is formed on the aggregates. All copolymers showed two populations (see size distribution curves depicted in Figure 7). QLBD1 formed a minor (in terms of scattering intensity) population of SCNs consisting of 3 nm nanoparticles and a major population consisting of aggregates of particles of 93 nm. Judging from the low scattered light intensity of the QLBD copolymers in water compared to LBD ones, the aggregates formed by QLBD copolymers have lower mass and may be looser. QLBD2 forms a slightly smaller percentage of SCNs and a slightly larger one, but in the size of 5 nm, they are extremely small. The large aggregates formed are almost the same size to those of LBD2 and due to the lower mass should be looser. This loose aggregate formation is a consequence of random monomer distribution along copolymer chains and the high hydrophilicity of QDMAEMA segments. The results of QLBD3 were notable. SCNs of 7 nm are present and in a larger percentage than the multichain aggregates of around 94 nm, being also of low density. The combination of high BzMA hydrophobic content and higher hydrophilicity of QDPMAEMA segments may be more favorable for SCN formation in this case. Notably, LBD1 and QLBD3 aggregates may show promising results for specific biomedical applications, as they consist mostly of SCNs. Coming to zeta potential results, as expected, all quaternized copolymers were positively charged, and once again, the DMAEMA ratio in polymer chains does not affect severely the positive charge (Table 2). As in LBD1 and QLBD1, the particles are less positively charged than LBD2 and QLBD2, respectively. This also happens in the hyperbranched copolymers synthesized by our group, where HB1 is less positively charged than HB2. Interestingly, the DMAEMA ratio in HB1 is greater than in HB2 [28].

**Figure 8:**
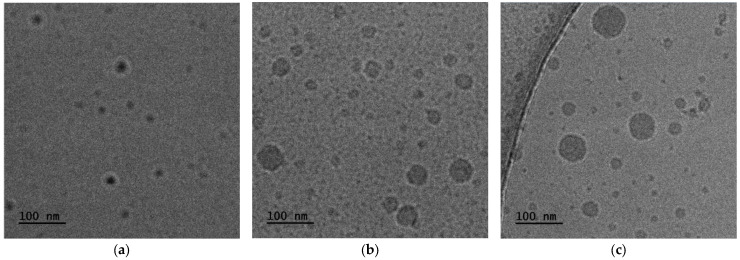
Cryo-TEM images from solutions of samples (**a**) LBD1, (**b**) LBD2, and (**c**) LBD3.

The copolymer aqueous solutions were also analyzed with cryo-TEM. The morphology of all amino copolymer aggregates investigated is that of spheres (globules). Measurements of 100 particles concluded with the average size presented below. Cry-TEM sizes of the SCNs formed by the LBD1 copolymer were small, with a mean size of 14 nm [30,31], and low contrast, verifying the observed intensity weighted size distributions in DLS measurements. In TEM measurements it is obvious that there is only one population of particles in this sample opposing the results of the DLS measurements (Figure 8a). This can be attributed to a combination of hydrophobic interactions counteracting electrostatic interactions between the nanoparticles, as DLS cannot identify the number of nanoparticles forming the aggregate. This may also indicate that the large aggregates observed in DLS may be aggregates/assemblies of SCNs and not a multichain nanoparticle. As for the LBD2 multichain, particles were observed with sizes ranging from 10 to 80 nm with an average size of 32 nm (Figure 8b); a few particles with sizes over 100 nm were also present. In the LBD3 copolymer solutions, sizes ranged from 10 to 115 nm, with an average size of 33 nm (Figure 8c). In both cases of LBD2 and LBD3, the nanoparticle size distribution seems high, but this is attributed to their high hydrophobic content. The high hydrophobic content affects their stability in aqueous solutions, and this also enhances aggregation. The aggregation is also observed in the DLS measurements where the scattered light intensity is greater than the one determined in LBD1, where aggregation did not occur. More images and the histogram of the particle size distributions can be found in Figures S5–S7.

**Figure 9:**
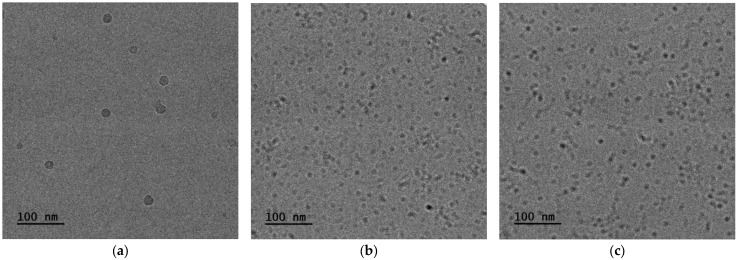
Cryo-TEM images from solutions of copolymers (**a**) QLBD1, (**b**) QLBD2, and (**c**) QLBD3.

The quaternized copolymer solutions exhibited a more complex morphology, characterized by binary particle populations that confirmed the initial DLS measurements. In sample QLBD1, two distinct fractions of spherical nanoparticles were identified: small, low-contrast particles in the size of 8–15 nm (Figure 9a) and higher-contrast particles ranging from 15–60 nm (Figure S8). Similarly, QLBD2 solutions displayed two populations in both DLS and cryo-TEM: the first consisted of spherical or near-spherical particles of 8–20 nm (Figure 9b), while the second comprised irregular or quasi-spherical aggregates of 30–200 nm (Figure S9). These larger structures are likely formed due to the copolymer’s higher hydrophobic content, though their irregular shapes made a definitive upper-size limit difficult to estimate. Lastly, QLBD3 featured spherical particles of 8–30 nm (Figure S10) alongside unique, rod-like structures with sizes ranging 20–60 nm in length, probably formed through the aggregation of smaller units (Figure 9c). The small fraction observed by cryo-TEM likely corresponds to the ~6 nm particles measured by DLS; the 1–2 nm discrepancy is likely due to a lack of sufficient electron density.

#### 3.4.2. Influence of Solution pH and Response to Temperature

**Figure 10:**
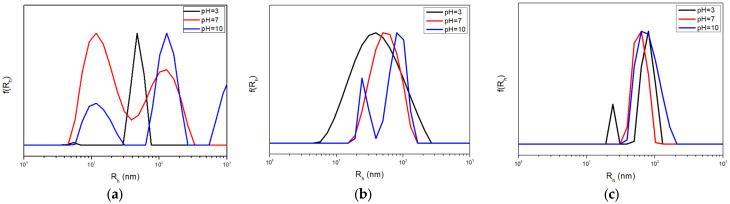
pH-responsive behavior of copolymers (**a**) LBD1, (**b**) LBD2 and (**c**) LBD3.

Copolymers containing DMAEMA monomers display interesting properties due to the known thermal and pH responsiveness of the PDMAEMA homopolymer. The pH-responsiveness is attributed to the protonation and deprotonation of the tertiary amine groups, which affects polymer hydrophilicity [24]. When the amine groups are deprotonated, the DMAEMA segments tend to be less hydrophilic. The thermal response is connected to the LCST of PDMAEMA, where the weak hydrogen bonding between the amine groups and water molecules brakes at a higher temperature, also affecting homopolymer hydrophilicity [24]. The behavior for sample LBD1 at different pHs is depicted in Figure 10a, starting from solutions at neutral pH (pH = 7, red curve) by decreasing and increasing pH by adding HCl or NaOH solutions, respectively. As the amine groups are protonated in acidic pH, the nanoparticles change their tendency to aggregate. In this case two things happen: amine groups protonate and copolymer chains become more soluble, resulting in disaggregation of the multichain nanoparticles, forming smaller and more well defined ones of 48 nm. The second thing that might occur is that in the SCNs, the trapped DMAEMA segments start to emerge in the corona, and the hydrophobic segments of multiple chains aggregate, merging into multichain nanoparticles (the small peak at ca. 6 nm shows a small portion of SCNs remaining). The disaggregation results from the higher hydrophilicity of protonated DMAEMA segments and the higher positive charge creates repulsion of the particles. At pH 10 as the amine groups deprotonate and the DMAEMA domains tend to become less hydrophilic, aggregation of initial aggregates occurs. For copolymer LBD2 (Figure 10b), in acidic pH disaggregation occurs. This can be concluded from the significant decrease in the scattered light intensity and the decrease in average nanoparticle size (Table 2). A rise in the size polydispersity index can be observed, which is attributed to particle rearrangement when they disaggregate due to their higher positive charge. Two separate populations cannot be identified, probably because their R_h_ values are close, and a broad size distribution peak is observed. As for the basic pH, probably aggregation occurs (scattered intensity increases, Table 2) as DMAEMA becomes less hydrophilic, forming two populations of particles with distinct sizes. A notable result is that LBD1 particles have a higher cationic charge at pH = 3 than LBD2, which proves that the DMAEMA segments are sterically hindered from BzMA at pH = 7. As the amine groups in LBD3 get protonated in acidic pH values, two populations of aggregates appear. The particles start to repel each other due to the high positive charge. A small number of particles are able to disaggregate due to the repulsion, forming the small population. The vast number of aggregates swell but still the developed charge is not high enough to completely disaggregate them. As pH becomes alkaline, the DMAEMA domains become less hydrophilic, aggregation occurs, and the copolymer particles start to precipitate. This can be concluded also by the significant drop in scattered intensity and by the rise in R_h_. The negative charge in zeta potential measurements displayed in Table 2 for all copolymer solutions at basic pH probably derives from the deprotonation of the -COOH groups of the CTA present at the copolymer chain ends, which should be located close to the surface of the copolymer aggregates. Lastly, OH^−^ ion absorption on the particle surface may take place, attributing negative charge to the particles observed in pH = 10 solutions [24,28].

**Figure 11:**
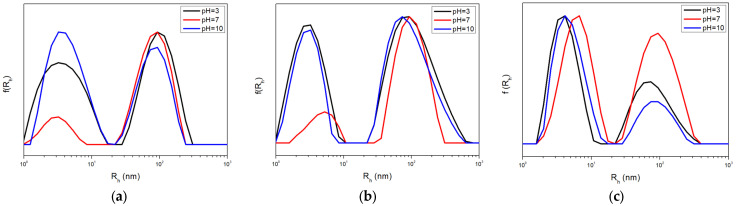
pH-responsive behavior of copolymers (**a**) QLBD1, (**b**) QLBD2 and (**c**) QLBD3.

After quaternization of the DMAEMA segments, the pH-responsiveness seems to degrade, as amine group protonation/deprotonation is not possible. With this chemical reaction, the modification is almost quantitative, which results in the presence of a minor amount of unmodified amine groups. Investigating if this amount is enough in order to have a pH-responsive copolymer system might be interesting. QLBD1 zeta potential values remain constant in the three pH values investigated (Table 2), which means that quaternization reached very high convention. Interesting results come from the disaggregation of the particles in SCNs depicted in Figure 11a. In acidic and alkaline pH values, because of pH adjustment with HCl and NaOH, the conformational changes are due to changes in the ionic strength of the copolymer solutions. In Figure 11b, a similar response of QLBD2 copolymer solutions can be seen. The zeta potential values show minor changes. In Figure 11c, once again, disaggregation occurs as the pH is adjusted to different values. In acidic pH values QLBD3 solutions show a minor rise in the scattered light intensity; this is attributed to a tighter particle conformation, hence the smaller R_h_ value. Once again, the zeta potential values remain almost constant, indicating the quantitative conversion of amine groups to quaternized amine groups.

**Figure 12:**
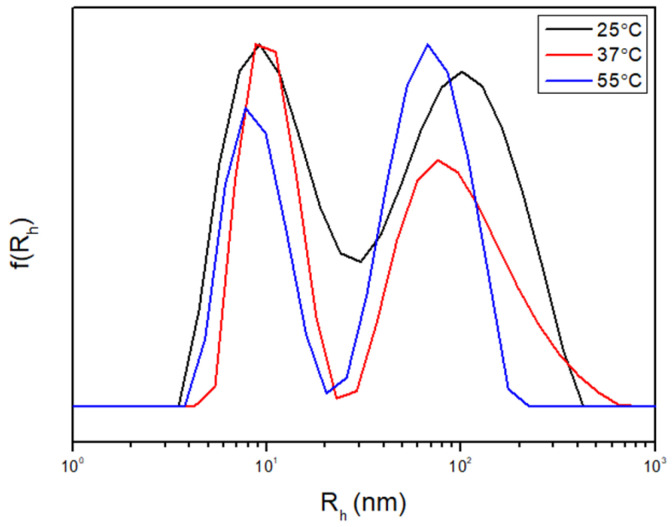
Size distributions from CONTIN analysis of DLS data from the thermal response of LBD1 copolymer solution.

All amine copolymer solutions at neutral pH were investigated for their thermal response. Only LBD1 showed changes as expected due to the high DMAEMA content; results are displayed in Figure 12. The LCST of PDMAEMA is in the range of 35–45 °C; this range is dictated mainly by the molecular weight of PDMAEMA (and correspondingly to the polymer chain length). In a statistical copolymer this value can differ substantially due to the comonomer distribution in the polymeric chain. In our case, at 37 °C the larger population of particles started to disaggregate into SCNs. Most of the particles measured in DLS were SCNs; a small number of the SCNs, due to the rupture of hydrogen bonds, became less hydrophilic, forming smaller aggregates of R_h_ = 76 nm. As the temperature increased to 55 °C, the copolymer chains became even less hydrophilic; therefore, SCNs aggregated, forming even smaller aggregates of R_h_ = 67 nm and probably more compact ones. Across the temperatures, the majority of the particles in these solutions were in the form of SCNs.

#### 3.4.3. Ionic Strength Effects and Interaction of the Copolymers with Fetal Bovine Serum

**Figure 13:**
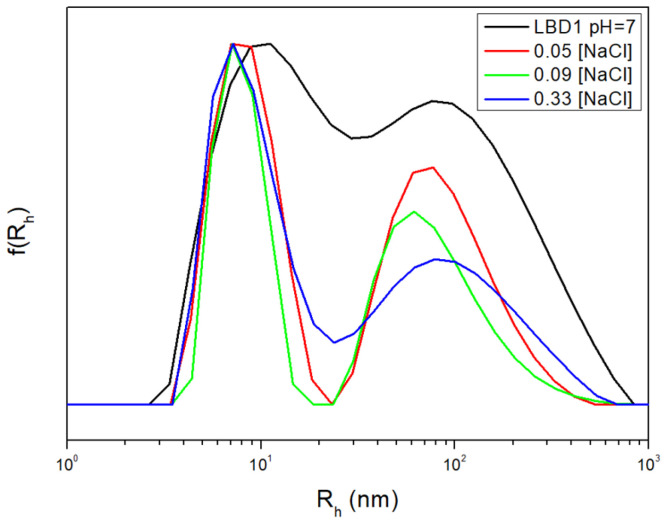
Size distributions from CONTIN analysis of DLS data for the ionic strength variation of LBD1 aqueous solutions at neutral pH.

The solutions of LBD1 and the quaternized copolymer were investigated for possible ionic strength effects on their self-assembly properties since they contain the larger number of ionized amine groups. The ionic strength effects are related to the ability of the copolymers to change their self-assembly in the presence of salt, and these properties are important for biological and industrial applications of the present novel copolymers. The presence of salt modulates intermolecular interactions, altering the effective surface charge of particles, and may result in different chain conformations and macromolecular assemblies. The copolymers were investigated in two pH values: in neutral pH, where the amine groups are partially protonated due to the DMAEMA pKa ≈ 7.4, and in pH = 3, where the amine groups are fully protonated. Three salt concentrations were utilized: 0.05, 0.09, and 0.33 M of NaCl and 0.1 M in a solution of 1 mg/mL polymer in distilled water. In alkaline pH values, the deprotonated amine groups are less amenable to interacting with ions; therefore, such measurements were not conducted. In acidic pH for LBD1 solutions, a minor disaggregation occurred as the salt concentration increased. This can be attributed to the amine groups’ electrostatic interactions playing a significant role in the self-assembly of the preformed nanoparticles. In neutral pH values, the electrostatic interactions are weaker; therefore, the aggregation of the particles is larger. This can be confirmed with two results, the first being the one that scattered intensity remains almost constant, and the second being the decrease in the measured hydrodynamic radius of the particles. In Figure 13, the reduction in scattering from the large population of the aggregated SCNs and the rise in the disaggregated SCNs are depicted. For LBD2, at pH = 3, as the salt concentration increases, a small percentage of particles disaggregate. This disaggregation is about 10% of the larger population (based on intensity). In pH = 7, contrary to the LBD1 in pH = 7, the single population is more stable, and disaggregation does not occur. Only a small percentage of ca. 6% disaggregation into SCNs of R_h_ = 11 nm is observed. For LBD3, at pH = 3, as the salt concentration increases, the copolymer chains start to precipitate. This can be concluded from scattered intensity gradually decreasing until it almost becomes a fourth of that before salt addition. In pH = 7, the aggregates are more stable, and as the salt concentration increases, scattered intensity remains essentially constant, and there is only a minor decrease in scattered light intensity, which can be attributed to the precipitation of the small population of particles emerging at 0.09 M NaCl concentration. All these results can be found in Supplementary Information in Figures S11–13.

**Figure 14:**
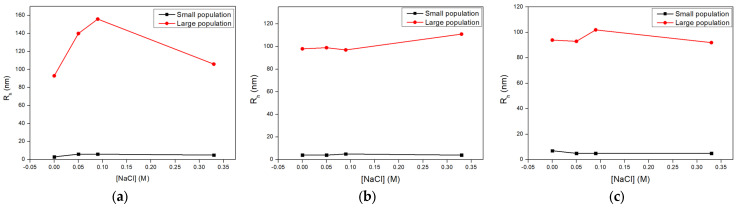
Ionic strength effect on quaternized copolymer solutions and the variation in R_h_ values determined from CONTIN analysis of DLS data for (**a**) QLBD1, (**b**) QLBD2, (**c**) QLBD3.

The quaternized counterparts of the copolymers did not exhibit any significant pH-responsiveness. Therefore, the ionic strength of this series of copolymers was investigated only under neutral pH conditions. As salt concentration increases, the majority of QLBD1 aggregated particles disaggregate, forming SCNs of 6 nm and a minor number of aggregated particles forming a large population in the range of 105–156 nm (see Supplementary Materials). The interesting part in these results is that the increase in salt concentration decreases the size of the larger aggregates, as depicted in Figure 14a. For sample QLBD2, the same response is noted. The aggregated particles start to disaggregate, forming SCNs of 5 nm, and the larger population remains constant until 0.33 M NaCl, where a small increase in R_h_ from 98 to 111 nm is seen. From the DLS measurements and the R_h_ cumulant values presented in the Supplementary Information (Figures S14–S16 and Table S1), the percentage of the aggregated particles is smaller than the SCNs. Lastly in QLBD3 solutions, the majority of aggregates start to form SCNs of 5 nm, as salt concentration increases, and the large population, which is the minority, remains almost constant at 92 nm. These results confirm that the quaternized copolymers do not respond to pH alterations in agreement with expectations.

**Figure 15:**
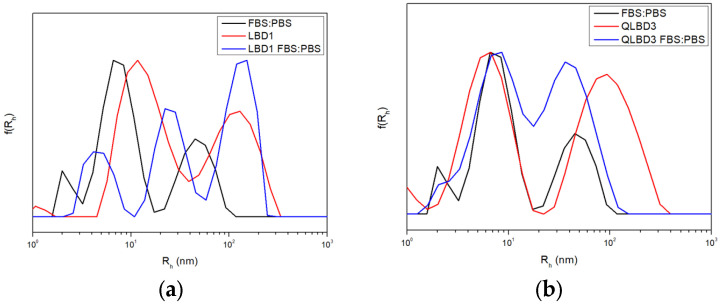
FBS:PBS interaction studies with (**a**) LBD1 and (**b**) QLBD3 from DLS measurements.

The fetal bovine serum in phosphate-buffered saline mimics the blood environment, since it includes proteins and salts at physiological levels. Studying the interactions of FBS:PBS with copolymer assemblies can give an insight into how these copolymer systems may behave in these biologically relevant conditions. As the proteins in this serum are negatively charged, interactions in the positive corona of the micelles are expected. In LBD1 the interactions are prominent. The sample interacts with FBS:PBS, and it forms three copolymer-protein mixed populations, as depicted in Figure 15a. However, none of these exceed the micrometer scale, with the larger consisting of particles in the size of 153 nm. As the scattered light intensity increases, interactions are becoming more obvious. When serum is mixed with LBD2 and LBD3 solutions, precipitation occurs, as can be deduced from the reduction in the scattered intensity, thus forming micrometer-scale particles (Figures S17 and S18)—something that is not desirable for biomedical applications. As the quaternized counterparts from this series were utilized in an FBS:PBS environment, precipitation was expected due to their high positive charge. In mixed QLBD1 solutions, where the quaternized amine groups were the most common and had the highest positive charge, three populations were formed. The smallest consisted of particles with R_h_ values of 9 nm, the second being 80 nm, and the third being 1780 nm. From these results, we can conclude that precipitation is occurring (Figure S19). QLBD2 showed similar interactions to QLBD1, and the three populations consisting of 4 nm, 30 nm, and 494 nm are depicted in Figure S20. In Figure 15b, the QLBD3 interaction with FBS:PBS is depicted. Interestingly, this sample forms two populations, the smaller consisting of 8 nm aggregates and the larger consisting of 40 nm. FBS:PBS contains mainly albumin, which is amphiphilic and may cause disaggregation of the amphiphilic copolymer aggregates by interaction with their hydrophobic domains. No micrometer scale aggregation was observed.

## 4. Conclusions

Amphiphilic statistical copolymers of the P(BzMA-co-DMAEMA) type with three different compositions were successfully synthesized via a one-step RAFT polymerization scheme, as was proven by SEC, ^1^H-NMR, and FTIR molecular characterization. Their chemical modification with methyl iodide produced cationic amphiphilic macromolecules. The self-assembly of both families of amphiphilic copolymers was studied in aqueous solutions using cryo-TEM and light-scattering techniques. Amine-based LBD copolymers show response to solution pH, while QLBD copolymers’ assembly is influenced mainly by solution ionic strength. Interestingly, the LBD1 and QLBD1-QLBD3 copolymers are forming SCNs, which are ultra-small in some cases, making them interesting candidates for surpassing tough biological barriers. It was noticed that by adjusting the pH and ionic strength of these copolymer aqueous solutions, the formation of SCNs was enhanced. Additionally, for the amino copolymers, disaggregation was favorable in acidic pH values, while aggregation was favorable at basic pH. As anticipated, the quaternized counterparts did not show pH responsiveness. Ionic strength measurements of these copolymers indicated that disaggregation was favored by salt concentration increase only in the samples where SCNs were present. Copolymer–FBS:PBS interaction studies by DLS showed that the strongly positively charged QLBD copolymers may form aggregates with serum proteins, sometimes reaching microscale structures followed by precipitation. These experiments proved that LBD1 and QLBD3 copolymers may be the most promising copolymer for biomedical and biotechnological applications due to simple synthesis and nanoparticle characteristics, including drug and gene delivery, bioimaging and enzyme immobilization, and nanostructuring by the use of additional functional components. Overall, our results enrich existing knowledge on random copolymer solution self-assembly. We believe that this study shows that amphiphilic random/statistical copolymers can have a rich behavior in aqueous media, which can be tuned by their chemical composition and their tunable solvophilicity.

## Figures and Tables

**Table 1:** Molecular characteristics of the synthesized P(BzMA-co-DMAEMA) amphiphilic copolymers.

Sample	M_w_^a^ (×10^4^ gmol^−1^)	M_w_/M_n_^a^	BzMA ^b^ (wt%)	DMAEMA ^b^ (wt%)
LBD1	3.7	1.53	23	77
LBD2	3.8	1.73	46	54
LBD3	3.3	1.72	63	37

^a^: determined by SEC, ^b^: determined by ^1^H-NMR.

**Table 2:** Results of DLS and ELS measurements for the linear amino copolymers and their quaternized counterparts.

Sample	pH	Int_90_ (Kcps)	R_h_ (nm)	PDI	Zeta Potential (mV)
LBD1	3	651	6/48	0.21	+70 ± 1.2
	7	337	9/101	0.48	+30 ± 1
	10	3640	12/129/1584	0.48	−42 ± 1
LBD2	3	160	40	0.45	+65 ± 0.5
	7	25,700	53	0.22	+43 ± 1.3
	10	37,400	24/80	0.27	−32 ± 0.8
LBD3	3	289,500	24/78	0.22	+52.1 ± 1
	7	130,000	60	0.17	+27 ± 1.5
	10	65,500	78	0.22	−38 ± 1.8
QLBD1	3	35	4/96	0.50	+49 ± 4
	7	22	3/93	0.63	+49 ± 2
	10	34	4/81	0.48	+48
QLBD2	3	73	3/103	0.50	+58 ± 13
	7	52	5/98	0.60	+ 67 ± 6
	10	70	3/93	0.52	+47 ± 4
QLBD3	3	110	4/78	0.48	+34 ± 3
	7	72	7/94	0.50	+37 ± 1
	10	70	5/88	0.46	+33 ± 1

## Data Availability

Data produced in this study are included in the manuscript and the Supplementary Materials.

## Supplementary Materials

The following supporting information can be downloaded at https://www.mdpi.com/article/10.3390/polym18111278/s1, Figure S1: ^1^H-NMR spectrum of LBD2 in CDCl_3_; Figure S2. ^1^H-NMR spectrum of LBD3 in CDCl_3_; Figure S3. ATR-FTIR spectra of LBD2 and QLBD2 indicating the successful formation of the quaternized copolymer; Figure S4. ATR-FTIR spectra of LBD3 and QLBD3 indicating the successful formation of the quaternized copolymer; Figure S5. (a) Cryo-TEM image of LBD1 and (b) the corresponding particle size distribution; Figure S6. (a) Cryo-TEM image of LBD2 and (b) the corresponding particle size distribution; Figure S7. (a) Cryo-TEM image of LBD3 and (b) the corresponding particle size distribution; Figure S8. Cryo-TEM image of QLBD1 showing high contrast nanoparticles; Figure S9. Cryo-TEM image of QLBD2 showing irregular nanoparticles. The larger, more globular ones show some internal structuration; Figure S10. Cryo-TEM image of QLBD3 showing spherical uniform particles; Figure S11. Size distributions from CONTIN analysis of DLS data for different ionic strength values of LBD1 aqueous solutions at pH = 3; Figure S12. Size distributions from CONTIN analysis of DLS data for different ionic strength values of LBD2 aqueous solutions at (a) pH = 3 and (b) pH = 7; Figure S13. Size distributions from CONTIN analysis of DLS data for different ionic strength values of LBD3 aqueous solutions at (a) pH = 3 and (b) pH = 7; Figure S14. Size distributions from CONTIN analysis of DLS data for different ionic strength values of QLBD1 aqueous solutions at neutral pH; Figure S15. Size distributions from CONTIN analysis of DLS data for different ionic strength values of QLBD2 aqueous solutions at neutral pH; Figure S16. Size distributions from CONTIN analysis of DLS data for different ionic strength values of QLBD3 aqueous solutions at neutral pH; Table S1. DLS results for quaternized copolymers aqueous solutions of different ionic strength; Figure S17. Size distributions from CONTIN analysis of DLS data illustrating LBD2 aggregates interaction with FBS:PBS media; Figure S18. Size distribution from CONTIN analysis of DLS data illustrating LBD3 aggregates interaction with FBS:PBS media; Figure S19. Size distributions from CONTIN analysis of DLS data illustrating QLBD1 aggregates interaction with FBS:PBS media; Figure S20. Size distributions from CONTIN analysis of DLS data illustrating QLBD2 aggregates interaction with FBS:PBS media.
