# Supplementary material for: Linear Amphiphilic P(BzMA-co-DMAEMA) Statistical Copolymers: Synthesis via RAFT Polymerization and Formation of Nanoassemblies in Aqueous Media
Source: Polymers (Basel). 2026 May 22;18(11):1278. doi: 10.3390/polym18111278 (PMC13259143; doi:10.3390/polym18111278)
Supplement: Supplementary file 1 [file polymers-18-01278-s001.zip › polymers-4317812-supplementary.pdf]

Linear amphiphilic P(BzMA-co-DMAEMA) statistical copolymers: Synthesis via RAFT polymerization and formation of nanoassemblies in aqueous media

Stamatios Amarantos [1], Michaila Akathi Pantelaiou [1,2] , Aleksander Forys[3], Barbara Trzebicka[3] and Stergios Pispas [1,*]

1 Theoretical and Physical Chemistry Institute, National Hellenic Research Foundation, 48 Vassileos Constantinou Ave., 11635 Athens, Greece

2 Department of Chemistry, National and Kapodistrian University of Athens, Panepistimiopolis Zografou, 15771 Athens, Greece

3 Centre of Polymer and Carbon Materials, Polish Academy of Sciences, M. Curie-Sklodowskiej 34, Zabrze, 41-819, Poland

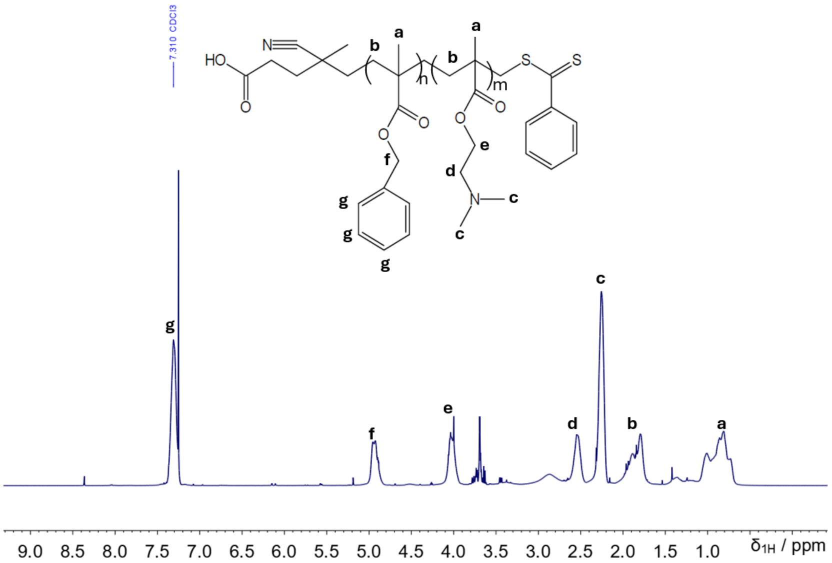

Figure S1. ^{1}H-NMR spectrum of LBD2 in $CDCl_3$.

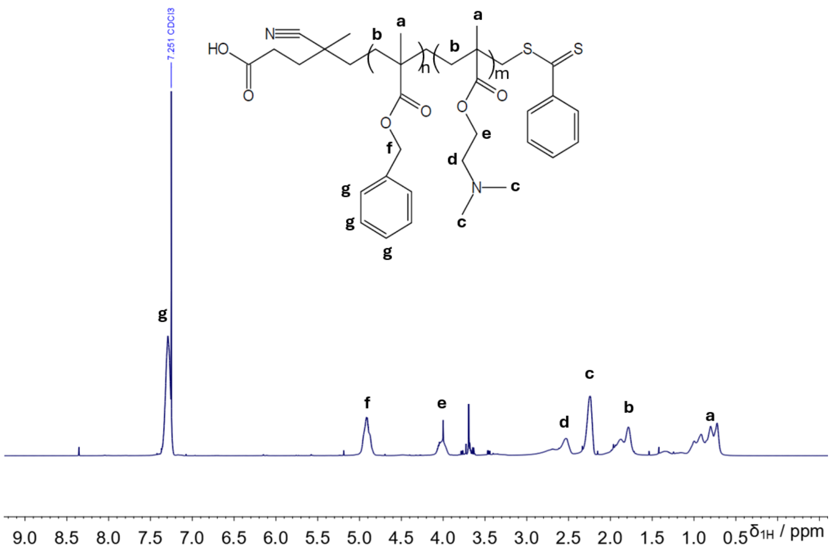

Figure S2. ^{1}H-NMR spectrum of LBD3 in $CDCl_3$.

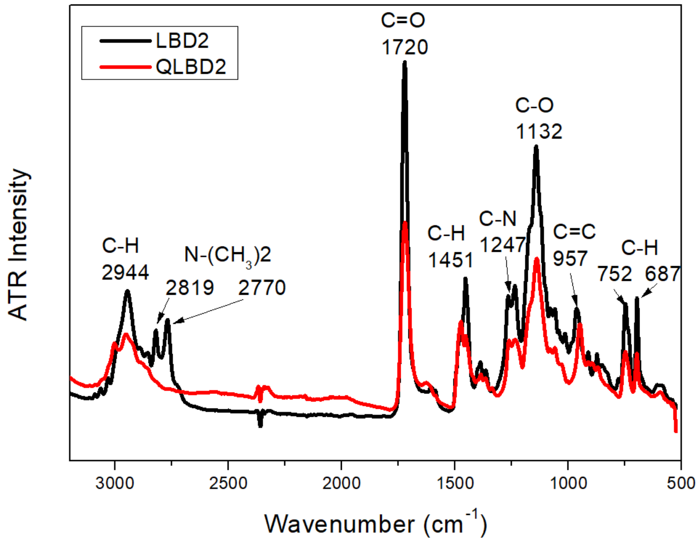

Figure S3. ATR-FTIR spectra of LBD2 and QLBD2 indicating the successful formation of the quaternized copolymer.

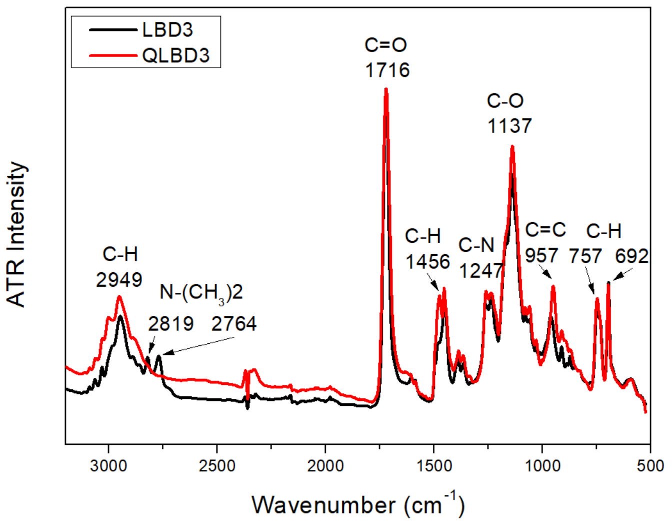

Figure S4. ATR-FTIR spectra of LBD3 and QLBD3 indicating the successful formation of the quaternized copolymer.

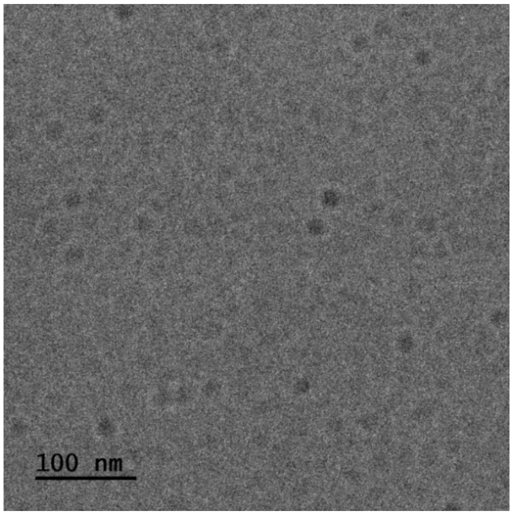

(a)

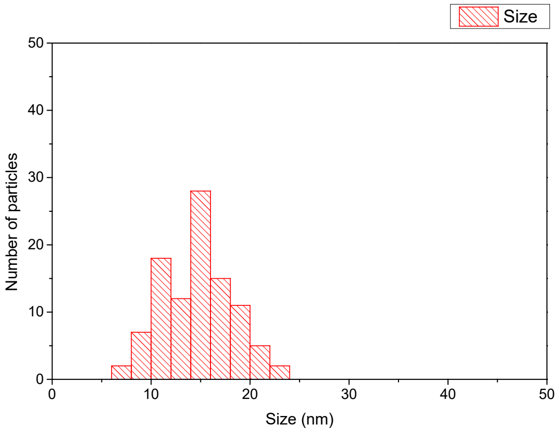

(b)

Figure S5. (a) Cryo-TEM image of LBD1 and **(b)** the corresponding particle size distribution.

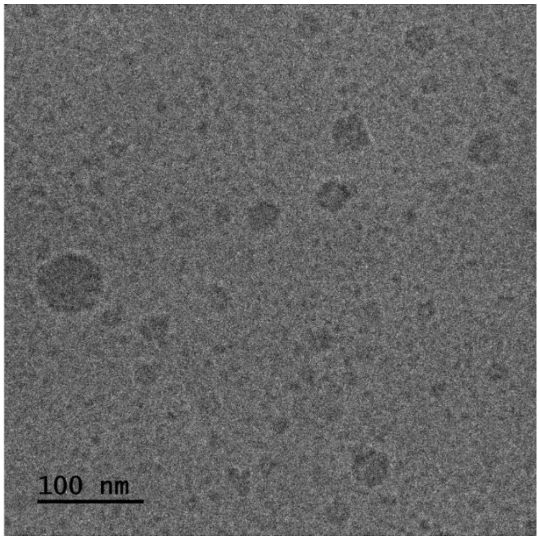

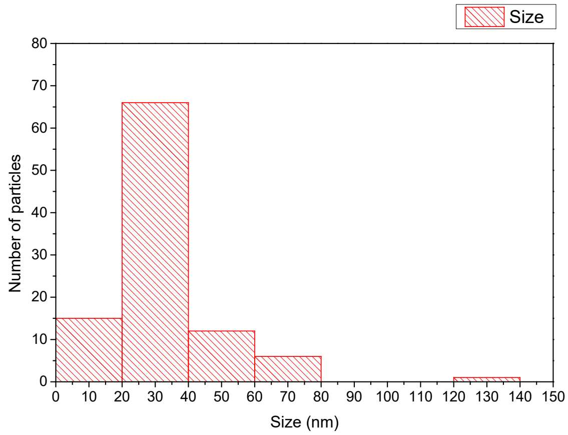

(a) **(b)**

Figure S6. (a) Cryo-TEM image of LBD2 and **(b)** the corresponding particle size distribution.

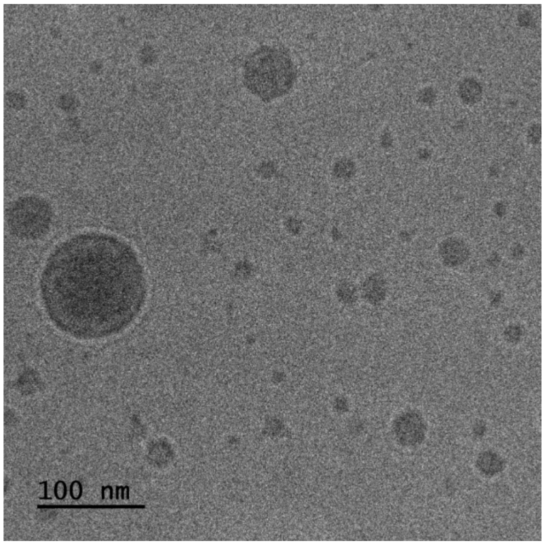

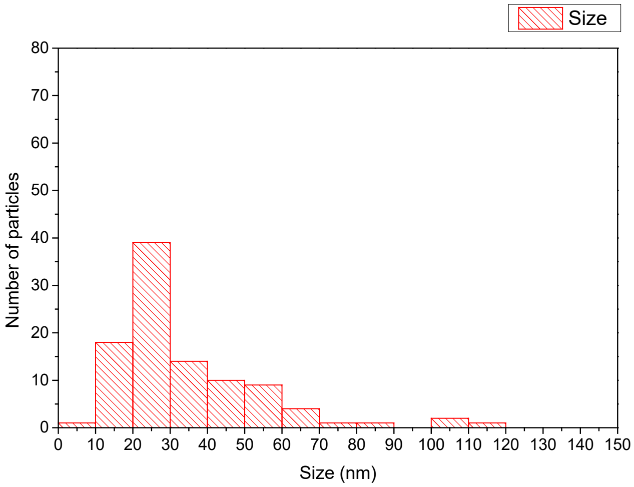

(a) **(b)**

Figure S7. (a) Cryo-TEM image of LBD3 and **(b)** the corresponding particle size distribution.

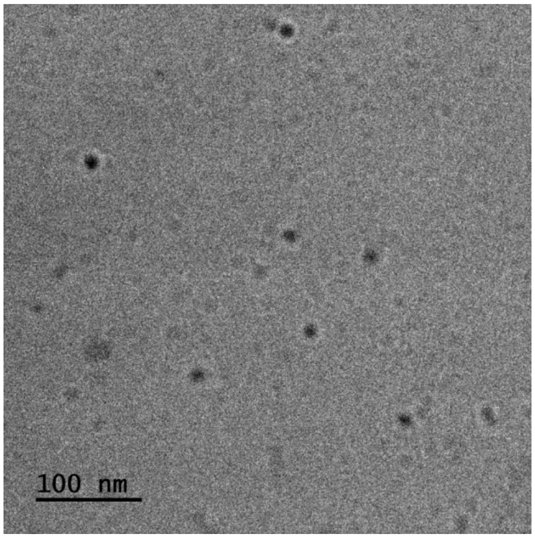

Figure S8. Cryo-TEM image of QLBD1 showing high contrast nanoparticles.

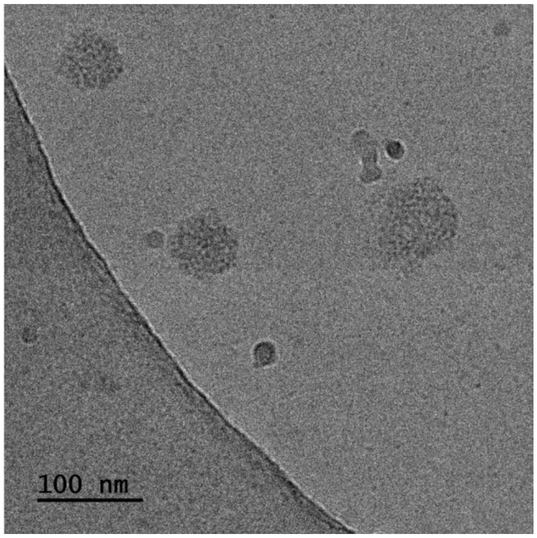

Figure S9. Cryo-TEM image of QLBD2 showing irregular nanoparticles. The larger, more globular ones show some internal structuration.

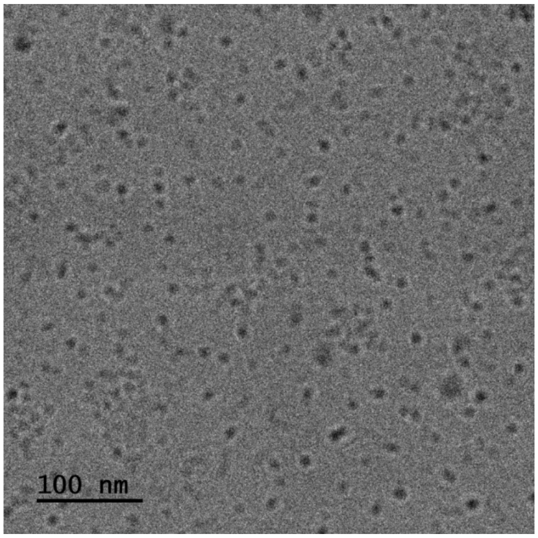

Figure S10. Cryo-TEM image of QLBD3 showing spherical uniform particles.

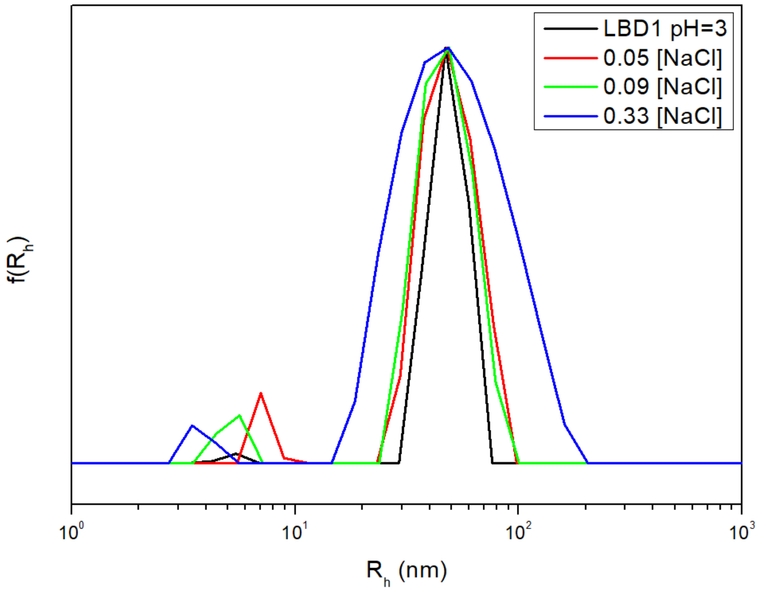

Figure S11. Size distributions from CONTIN analysis of DLS data for different ionic strength values of LBD1 aqueous solutions at pH=3.

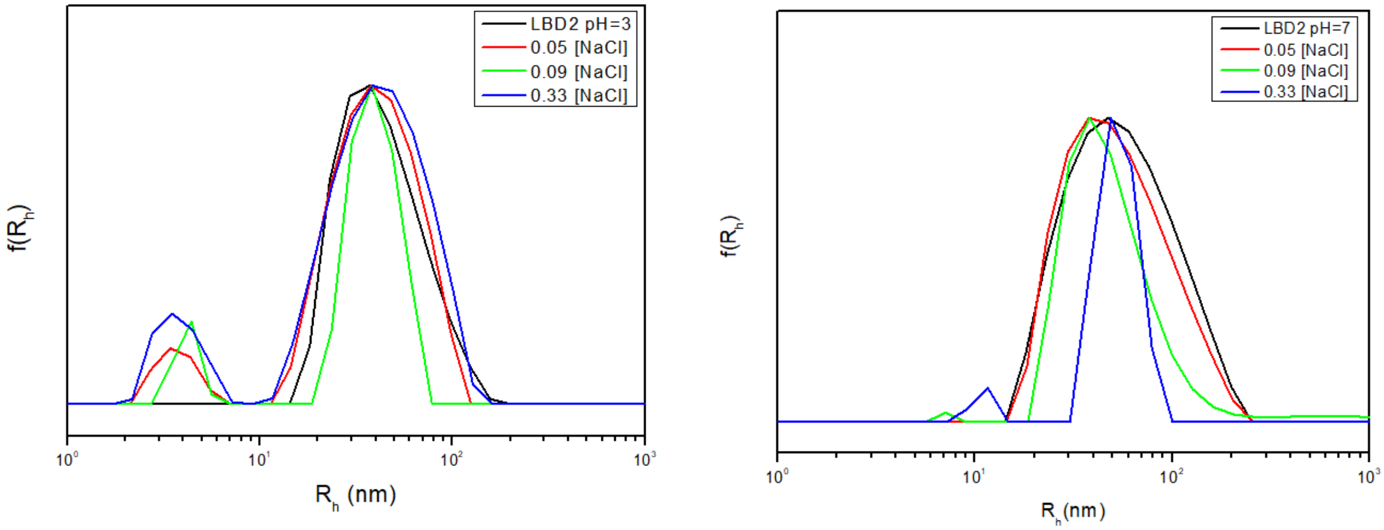

(a) **(b)**

Figure S12. Size distributions from CONTIN analysis of DLS data for different ionic strength values of LBD2 aqueous solutions at **(a)** pH=3 and **(b)** pH=7.

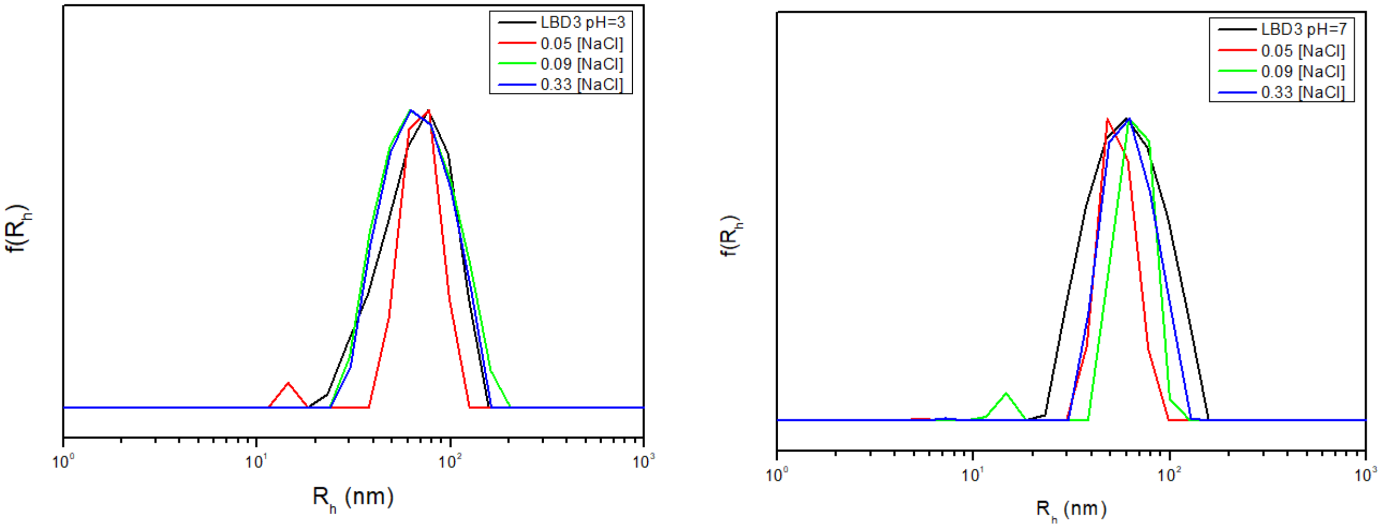

(a) **(b)**

Figure S13. Size distributions from CONTIN analysis of DLS data for different ionic strength values of LBD3 aqueous solutions at **(a)** pH=3 and **(b)** pH=7.

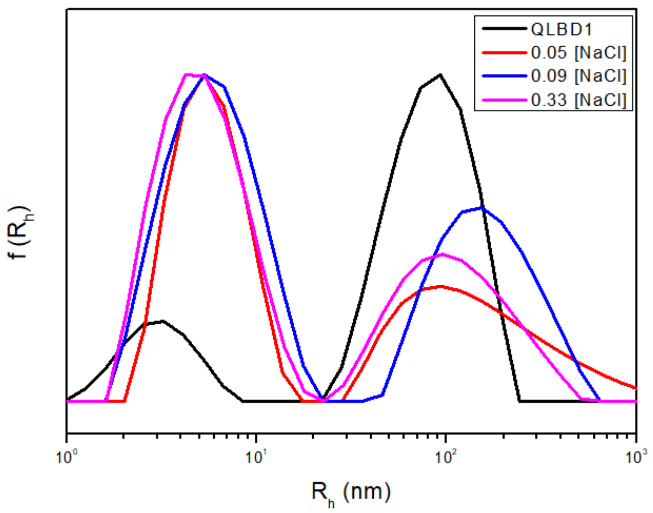

Figure S14. Size distributions from CONTIN analysis of DLS data for different ionic strength values of QLBD1 aqueous solutions at neutral pH.

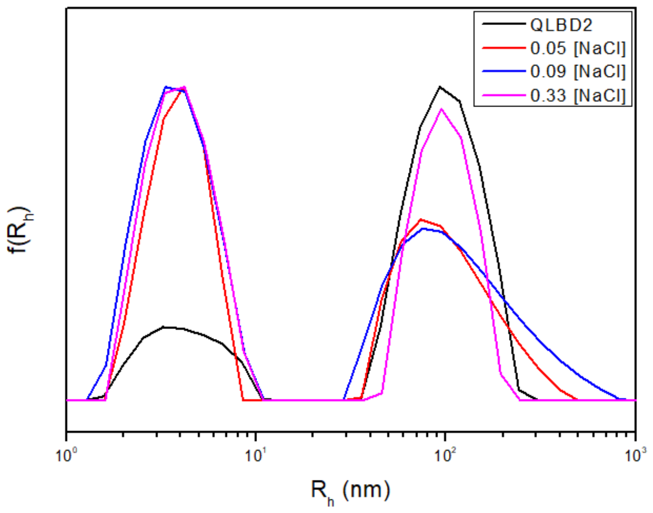

Figure S15. Size distributions from CONTIN analysis of DLS data for different ionic strength values of QLBD2 aqueous solutions at neutral pH.

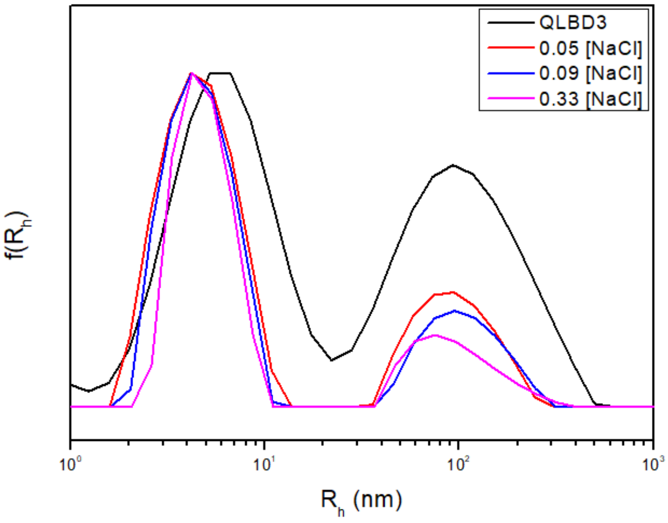

Figure S16. Size distributions from CONTIN analysis of DLS data for different ionic strength values of QLBD3 aqueous solutions at neutral pH.

Table S1. DLS results for quaternized copolymers aqueous solutions of different ionic strength

Sample	pH	[NaCl] (M)	Int_{90} (Kcps)	$R_{h\ cum}$ (nm)	PDI	$R_{hcontin}$ (nm)
QLBD1	7	0	22	34	0.63	3/93
		0.05	45	8	0.53	6/140
		0.09	48	9	0.5	6/156
		0.33	48	7	0.52	5/106
QLBD2	7	0	52	33	0.60	5/98
		0.05	85	9	0.50	4/99
		0.09	86	9	0.46	4/97
		0.33	82	9	0.46	4/111
QLBD3	7	0	72	11	0.50	7/94
		0.05	115	6	0.45	5/93
		0.09	119	6	0.43	5/102
		0.33	138	6	0.4	5/92

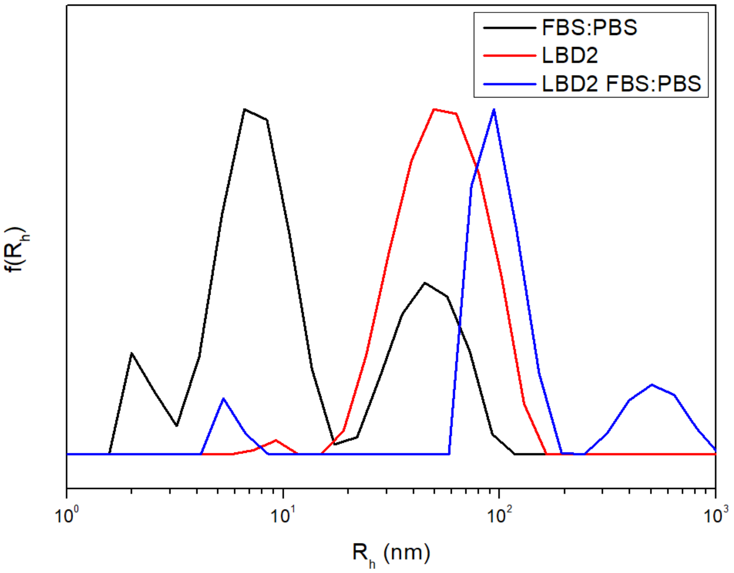

Figure S17. Size distributions from CONTIN analysis of DLS data illustrating LBD2 aggregates interaction with FBS:PBS media.

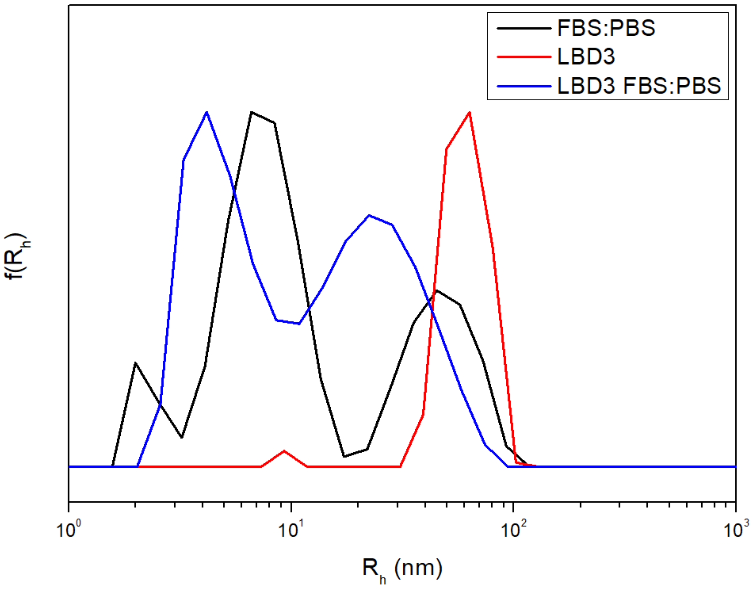

Figure S18. Size distribution from CONTIN analysis of DLS data illustrating LBD3 aggregates interaction with FBS:PBS media.

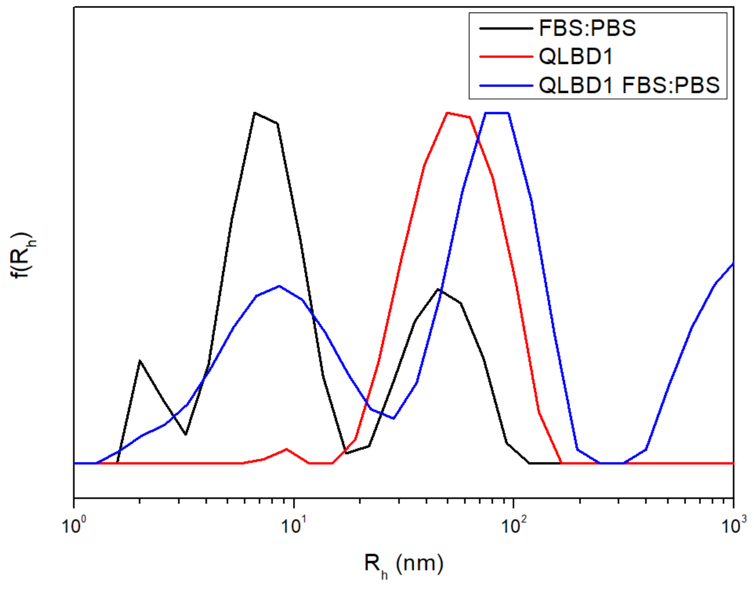

Figure S19. Size distributions from CONTIN analysis of DLS data illustrating QLBD1 aggregates interaction with FBS:PBS media.

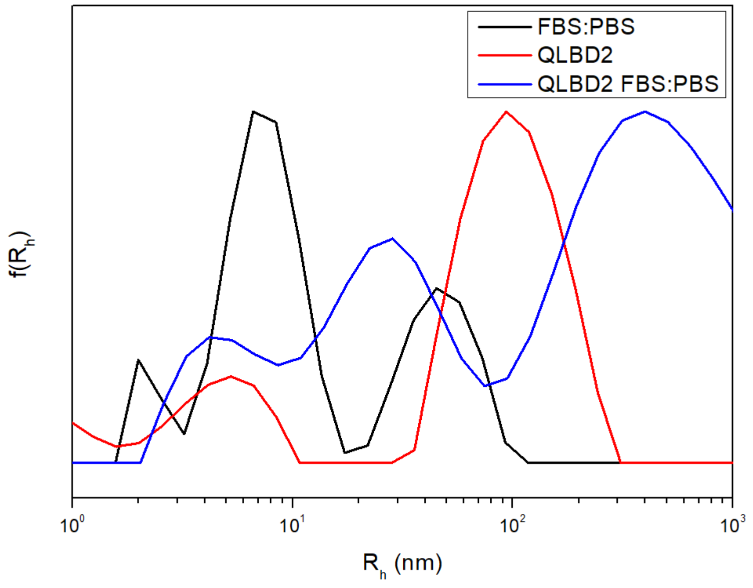

Figure S20. Size distributions from CONTIN analysis of DLS data illustrating QLBD2 aggregates interaction with FBS:PBS media.
